# Supplementary material for: Exploring the potential of artificial intelligence in individualized cognitive training: A systematic review
Source: PLoS One. 2025 Jun 17;20(6):e0316860. doi: 10.1371/journal.pone.0316860 (PMC12173387; doi:10.1371/journal.pone.0316860)
Supplement: S1 Text: Appendix — (PDF) [file pone.0316860.s001.pdf]

Supporting information

S1 Appendix. For the evaluation of potential bias within each controlled trial incorporated in this comprehensive review, we initially employed the Cochrane checklist to ascertain the underlying study design of each individual study. Subsequently, with regard to each dimension outlined in Table 6, a grading scale ranging from 0 (not applicable) to 3 (adequately addressed) was employed to assign scores. In instances where specific criteria were not explicitly stated, a grade of 1 was attributed. In situations involving crossover studies, a score of 2 was designated for the criterion pertaining to participant similarity between the control and intervention groups. Furthermore, for studies utilizing a crossover design, a score of 2 was ascribed to the concealment method criterion. In cases where researchers employed handcrafted questionnaires despite the existence of standardized alternatives, the assigned grade was automatically reduced by 1. The ultimate assessment presented in this table is established through the following categorizations: studies scoring 0 criteria as poorly addressed are denoted as (++), while those with 1 to 3 criteria marked as poorly addressed receive a rating of (+). Conversely, studies where more than 3 criteria are deemed inadequately addressed are indicated as (-), and non-comparative studies are indicated with (- -).

Identified Studies All identified studies are presented in table 6.

Table 6. List of identified studies

id_number	Reference	Title	Date
1	García-Rudolph and Gibert [12]	A data mining approach to identify cognitive NeuroRehabilitation Range in Traumatic Brain Injury patients	2014
2	Fermé et al. [97]	AI-Rehab: A Framework for AI Driven Neurorehabilitation Training - The Profiling Challenge	2020
3	Xu et al. [98]	Personalized Serious Games for Cognitive Intervention with Lifelog Visual Analytics	2018
4	Reidy et al. [99]	Facial Electromyography-based Adaptive Virtual Reality Gaming for Cognitive Training	2020
5	Kitakoshi et al. (a) [100]	Cognitive Training System for Dementia Prevention Using Memory Game Based on the Concept of Human-Agent Interaction	2015
6	Kitakoshi et al. (b) [101]	A Study on Coordination of Exercise Difficulty in Cognitive Training System for Older Adults, study-1	2020
7	Kitakoshi et al. (b) [101]	A Study on Coordination of Exercise Difficulty in Cognitive Training System for Older Adults - study-2	2020
8	Rathnayaka et al. [102]	Cognitive Rehabilitation based Personalized Solution for Dementia Patients using Reinforcement Learning	2021
9	Shen and Xu [103]	Research on children's cognitive development for learning disabilities using recommendation method	2020
10	Sandeep et al. [104]	Application of Machine Learning Models for Tracking Participant Skills in Cognitive Training - study-1	2020
11	Sandeep et al. [104]	Application of Machine Learning Models for Tracking Participant Skills in Cognitive Training - study-2	2020
12	Wilms [105]	Using artificial intelligence to control and adapt level of difficulty in computed-based cognitive therapy	2011
13	Solana et al. [106]	Intelligent Therapy Assistant (ITA) for cognitive rehabilitation in patients with acquired brain injury	2014
14	Zini et al. [24]	Adaptive cognitive training with reinforcement learning	2022
15	Zedda et al. [107]	Towards Adaptation of Humanoid Robot Behaviour in Serious Game Scenarios using Reinforcement Learning	2022
16	Eun et al. [108]	Development and Evaluation of an Artificial Intelligence–Based Cognitive Exercise Game: A Pilot Study	2022
17	Tsiakas et al. [109]	Task Engagement as Personalization Feedback for Socially-Assistive Robots and Cognitive Training	2018
18	Book et al. [110]	Individualised computerised cognitive training for community-dwelling people with mild cognitive impairment: study protocol of a completely virtual, randomized, controlled trial	2022
19	Singh et al. [25]	Deep learning-based predictions of older adults' adherence to cognitive training to support training efficacy	2022

Excluded Studies All excluded studied in full text review phase are presented in table 7.

Table 7. Excluded Studies (full text review phase)

Title	Authors	Exclusion reason
Adaptive play: A prototype of a responsive children's videogame for greater inclusivity	Jones, Rhianne	Not a cognitive training
Screening for mild cognitive impairment through digital biomarkers of cognitive performance in games	Gielis, Karsten	Adaptive procedure is not detailed
Understanding robots' potential to facilitate piano cognitive training in older adults with mild cognitive impairment	Mois, George; Collete, Bailey A.; Renzi-Hammond, Lisa M.; Boccanfuso, Laura; Ramachandran, Aditi; Gibson, Paul; Emerson, Kerstin G.; Beer, Jenay M.	The adaptive procedure adheres to a "unique trajectory design" (e.g similar to staircase algorithms)
Adaptive vs. non-adaptive cognitive training by means of a personalized App: a randomized trial in people with multiple sclerosis	Pedullà, L.; Brichetto, G.; Tacchino, A.; Vassallo, C.; Zaratin, P.; Battaglia, M.A.; Bonzano, L.; Bove, M.	The adaptive procedure adheres to a "unique trajectory design" (e.g similar to staircase algorithms)
Can computerized cognitive training reverse the diagnosis of HIV-associated neurocognitive disorder? A research protocol	Vance, David E.; Fazeli, Pariya L.; Azuero, Andres; Wadley, Virginia G.; Jensen, Michael; Raper, James L.	The adaptive procedure adheres to a "unique trajectory design" (e.g similar to staircase algorithms)
A comparison of two personalization and adaptive cognitive rehabilitation approaches: A randomized controlled trial with chronic stroke patients	Faria, A.L.; Pinho, M.S.; Bermúdez I Badia, S.	The adaptive procedure adheres to a "unique trajectory design" (e.g similar to staircase algorithms)
Mapping differential responses to cognitive training using machine learning.	Rennie, Joseph P.; Zhang, Mengya; Hawkins, Erin; Bathelt, Joe; Astle, Duncan E.	Duplicated in the list
Mapping differential responses to cognitive training using machine learning	Rennie, Joseph P.; Zhang, Mengya; Hawkins, Erin; Bathelt, Joe; Astle, Duncan E.	Intelligent techniques are used as tools for a better outcome analysis (e.g., effect size analysis)

Continued on next page

Table 7. Excluded Studies (full text review phase)

Title	Authors	Exclusion reason
Self-regulation of the anterior insula: Reinforcement learning using real-time fMRI neurofeedback	Lawrence, Emma J.; Su, Li; Barker, Gareth J.; Medford, Nick; Dalton, Jeffrey; Williams, Steve C. R.; Birbaumer, Niels; Veit, Ralf; Ranganatha, Sitaram; Bodurka, Jerzy; Brammer, Michael; Giampietro, Vincent; David, Anthony S.	Intelligent techniques are used as tools for a better outcome analysis (e.g., effect size analysis)
Can machine learning approaches lead toward personalized cognitive training?	Shani, Reut; Tal, Shachaf; Zilcha-Mano, Sigal; Okon-Singer, Hadas	Adaptive procedure is not detailed
Pupillometer-based neurofeedback cognitive training: Optimizing task engagement to enhance learning in prodrome, first episode, and established psychosis	Choi, J.; Fiszdon, J.; Stevens, M.; Haber, L.; Pearlson, G.	Non-peer-reviewed papers, opinion pieces, or abstract conference papers
Personalized on-line (computer-based) cognitive training (CogniFit) for patients with MS	Miller, A.; Shatil, E.	Non-peer-reviewed papers, opinion pieces, or abstract conference papers
Adaptive versus non-adaptive cognitive rehabilitation training based on working memory: Effects on people with multiple sclerosis	Pedullà, L.; Tacchino, A.; Vassallo, C.; Bonzano, L.; Battaglia, M.A.; Bove, M.; Brichetto, G.	Non-peer-reviewed papers, opinion pieces, or abstract conference papers
Understanding effects of cognitive rehabilitation under a knowledge discovery approach	García-Rudolph, A.; Gibert, K.	Intelligent techniques are used as tools for a better outcome analysis (e.g., effect size analysis)
Tailored and adaptive computerized cognitive training in older adults at risk for dementia: A randomized controlled trial	Bahar-Fuchs, A.; Webb, S.; Bartsch, L.; Clare, L.; Rebok, G.; Cherbuin, N.; Anstey, K.J.	The adaptive procedure is not detailed
Ontology-based personalization and modulation of computerized cognitive exercises	Quaglini, S.; Panzarasa, S.; Giorgiani, T.; Zucchella, C.; Bartolo, M.; Sinforiani, E.; Sandrini, G.	The adaptive procedure adheres to a ”unique trajectory design” (e.g similar to staircase algorithms)
PREVIRNEC: A cognitive telerehabilitation system based on virtual environments	Tost, D.; Grau, S.; Ferré, M.; García, P.; Tormos, J.M.; García, A.; Roig, T.	The adaptive procedure adheres to a ”unique trajectory design” (e.g similar to staircase algorithms)

Continued on next page

Table 7. Excluded Studies (full text review phase)

Title	Authors	Exclusion reason
A comparison of variable selection approaches for dynamic treatment regimes	Biernot, P.; Moodie, E.E.M.	Not a cognitive training
Machine beats human at sequencing visuals for perceptual-fluency practice	Sen, A.; Patel, P.; Rau, M.A.; Mason, B.; Nowak, R.; Rogers, T.T.; Zhu, X.	Adaptive procedure is not detailed
Development of perceptual training software for realizing high variability training paradigm and self adaptive training paradigm	Yang, R.; Nanjo, H.; Dantsuji, M.	The adaptive procedure adheres to a ”unique trajectory design” (e.g similar to staircase algorithms)
Personalized recommendation system for efficient integrated cognitive rehabilitation training based on bigdata	Kim, J.J.; Kim, Y.-J.; Lee, H.-M.; Lee, S.-H.; Chung, S.-T.	Not a cognitive training
Deciding the different robot roles for patient cognitive training	Andriella, A.; Alenyà, G.; Hernández-Farigola, J.; Torras, C.	Not a cognitive training
QiFei: Assisting to improve cognitive abilities for autism children using a mobile APP	Yi, C.; Ruan, F.; Gao, Y.; Hei, X.; Zhang, C.	The adaptive procedure adheres to a ”unique trajectory design” (e.g similar to staircase algorithms)
Predicting long-term outcome of Internet-delivered cognitive behavior therapy for social anxiety disorder using fMRI and support vector machine learning	Månsson, K.N.; Frick, A.; Boraxbekk, C.-J.; Marquand, A.F.; Williams, S.C.; Carlbring, P.; Andersson, G.; Furmark, T.	The adaptive procedure adheres to a ”unique trajectory design” (e.g similar to staircase algorithms)
Reh@City v2.0: A comprehensive virtual reality cognitive training system based on personalized and adaptive simulations of activities of daily living	Paulino, T.; Faria, A.L.; Bermudez Badia, S.	The adaptive procedure adheres to a ”unique trajectory design” (e.g similar to staircase algorithms)
Comparing adaptive cognitive training in virtual reality and paper-pencil in a sample of stroke patients	Faria, A.L.; Paulino, T.; Badia, S.B.I.	The adaptive procedure adheres to a ”unique trajectory design” (e.g similar to staircase algorithms)
A proposed automatic speech and sentiment recognition serious game for older adults with parkinson’s disease	Codreanu, I.-A.	Non-peer-reviewed papers, opinion pieces, or abstract conference papers
Serious games and ML for detecting MCI	Aljumaili, M.; McLeod, R.; Friesen, M.	Not a cognitive training
Effects of Personalized Cognitive Training with the Machine Learning Algorithm on Neural Efficiency in Healthy Younger Adults	Jeun, Y. J.; Nam, Y.; Lee, S. A.; Park, J. H.	The adaptive procedure adheres to a ”unique trajectory design” (e.g similar to staircase algorithms)
Possible evidence of near transfer effects after adaptive working memory training in persons with multiple sclerosis	í, S. F.; Tirado, A. S.; Sanchis-Segura, C.; Forn, C.	The adaptive procedure adheres to a ”unique trajectory design” (e.g similar to staircase algorithms)
Unifying framework for cognitive training interventions in brain aging	Turnbull, A.; Seitz, A.; Tadin, D.; Lin, F. V.	Adaptive procedure is not detailed

Continued on next page

Table 7. Excluded Studies (full text review phase)

Title	Authors	Exclusion reason
Leveraging technology to personalize cognitive enhancement methods in aging	Ziegler, D. A.; Anguera, J. A.; Gallen, C. L.; Hsu, W. Y.; Wais, P. E.; Gazzaley, A.	Adaptive procedure is not detailed
Effect of a multicomponent exercise program and cognitive stimulation (VIVIFRAIL-COGN) on falls in frail community older persons with high risk of falls: study protocol for a randomized multicenter control trial	nchez, J. L.; Udina, C.; n, A.; -Victor, M.; n, I.; Moral-Cuesta, D.; n-Epelde, I.; Ramon-Espinoza, F.; Latorre, M. S.; Idoate, F.; s, A.; nez, B.; Bonet, R. E.; Librero, J.; Casas-Herrero, Á.	The adaptive procedure adheres to a ”unique trajectory design” (e.g similar to staircase algorithms)
A Machine Learning Approach to Personalize Computerized Cognitive Training Interventions	Vladisauskas, M.; Belloli, L. M. L.; ndez Slezak, D.; Goldin, A. P.	Intelligent techniques are used as tools for a better outcome analysis (e.g., effect size analysis)
Impact of an individualized and adaptive cognitive intervention on working memory, planning and fluid reasoning processing in preschoolers from poor homes	Giovannetti, F.; Pietto, M. L.; Segretin, M. S.; Lipina, S. J.	The adaptive procedure adheres to a ”unique trajectory design” (e.g similar to staircase algorithms)
Toward Personalized Web-Based Cognitive Rehabilitation for Patients With Ischemic Stroke: Elo Rating Approach	Garcia-Rudolph, A.; Opisso, E.; Tormos, J. M.; Madai, V. I.; Frey, D.; Becerra, H.; Kelleher, J. D.; Bernabeu Guitart, M.; pez, J.	The adaptive procedure adheres to a ”unique trajectory design” (e.g similar to staircase algorithms)
Personalized Adaptive Training Improves Performance at a Professional First-Person Shooter Action Videogame	Neri, F.; Smeralda, C. L.; Momi, D.; Sprugnoli, G.; Menardi, A.; Ferrone, S.; Rossi, S.; Rossi, A.; Di Lorenzo, G.; Santarnecchi, E.	The adaptive procedure adheres to a ”unique trajectory design” (e.g similar to staircase algorithms)
		Continued on next page

Table 7. Excluded Studies (full text review phase)

Title	Authors	Exclusion reason
Personalized cognitive training: Protocol for individual-level meta-analysis implementing machine learning methods	Shani, R.; Tal, S.; Derakshan, N.; Cohen, N.; Enock, P. M.; McNally, R. J.; Mor, N.; Daches, S.; Williams, A. D.; Yiend, J.; Carlbring, P.; Kuckertz, J. M.; Yang, W.; Reinecke, A.; Beevers, C. G.; Bunnell, B. E.; Koster, E. H. W.; Zilcha-Mano, S.; Okon-Singer, H.	The adaptive procedure adheres to a ”unique trajectory design” (e.g similar to staircase algorithms)
Can Individualized-Targeted Computerized Cognitive Training Benefit Adults with HIV-Associated Neurocognitive Disorder? The Training on Purpose Study (TOPS)	Vance, D. E.; Fazeli, P. L.; Azuero, A.; Wadley, V. G.; Raper, J. L.; Ball, K. K.	The adaptive procedure adheres to a ”unique trajectory design” (e.g similar to staircase algorithms)
Applying Serious Games and Machine Learning for Cognitive Training and Screening: The COGNIPLAT Approach	Goumopoulos, Christos; Skikos, Georgios; Karapapas, Christos; Frounta, Maria; Koumanakos, Georgios	Non-peer-reviewed papers, opinion pieces, or abstract conference papers
An Environment to Collect Personal Memories of Older Adults and Use them to Personalise Serious Games with Humanoid Robots	Catricalà, B.; Ledda, M.; Manca, M.; Paternò, F.; Santoro, C.; Zedda, E.	The adaptive procedure adheres to a ”unique trajectory design” (e.g similar to staircase algorithms)
Functional improvement in chronic stroke patients when following a supervised home-based computerized cognitive training	Gil-Pagés, M.; Solana, J.; Sánchez-Carrión, R.; Tormos, J.M.; Enseñat-Cantallops, A.; García-Molina, A.	Non-peer-reviewed papers, opinion pieces, or abstract conference papers
Implementation of Clustering Techniques to Data Obtained from a Memory Match Game Oriented to the Cognitive Function of Attention	Orellana, M.; Acosta-Urigüen, M.-I.; García, R.R.	Non-peer-reviewed papers, opinion pieces, or abstract conference papers
Research on children’s cognitive development for learning disabilities using recommendation method	Shen, X.; Xu, C.	Duplicated in the list
CogRehab: A Personalized Digital Approach to Cognitive Rehabilitation	Mota, A.; Amorim, P.; Gabriel-Marques, A.; Serra, H.; Koc-Januchta, M.; Zagalo, H.; Sousa Santos, B.	The adaptive procedure adheres to a ”unique trajectory design” (e.g similar to staircase algorithms)

Continued on next page

Table 7. Excluded Studies (full text review phase)

Title	Authors	Exclusion reason
Machine Learning Techniques in Adaptive and Personalized Systems for Health and Wellness	Oyebode, Oladapo; Fowles, Jonathon; Steeves, Darren; Orji, Rita	Non-peer-reviewed papers, opinion pieces, or abstract conference papers
Efficacy of cogmed working memory training program in improving working memory in school-age children with and without neurological insults or disorders: A meta-analysis	Bharadwaj, Sneha V.; Yeatts, Paul; Headley, Johnna	Adaptive procedure is not detailed
SERIOUS GAMES WITH VIRTUAL REALITY AS A LEARNING PLATFORM FOR COGNITIVE TRAINING	Mocanu, Irina; Cramariuc, Oana; Cramariuc, Bogdan	Non-peer-reviewed papers, opinion pieces, or abstract conference papers
C-TESTER - SERIOUS GAMES FOR COGNITIVE TRAINING AND TESTING	Uller, Miroslav	Non-peer-reviewed papers, opinion pieces, or abstract conference papers
Predictive models for cognitive rehabilitation of patients with traumatic brain injury	Garcia-Rudolph, Alejandro; Garcia-Molina, Alberto; Munoz, Josep Maria Tormos	The adaptive procedure adheres to a ”unique trajectory design” (e.g similar to staircase algorithms)
AN INDIVIDUALIZED COGNITIVE INTERVENTION - DOES IT INCREASE THE EFFICACY OF BEHAVIORAL INTERVENTIONS FOR OBESITY	DELUCIA, JL; KALODNER, CR	The adaptive procedure adheres to a ”unique trajectory design” (e.g similar to staircase algorithms)
Ontology-Based Personalization and Modulation of Computerized Cognitive Exercises	Quaglini, Silvana; Panzarasa, Silvia; Giorgiani, Tiziana; Zucchella, Chiara; Bartolo, Michelangelo; Sinforiani, Elena; Sandrini, Giorgio	The personalization only consists in satisfying user preference (e.g visual features, content type, gaming component) without adapting the learning path)
NeuroCare-Personalization and Adaptation of Digital Training Programs for Mild Cognitive Impairments	Hardy, Sandro; Reuter, Christian; Goebel, Stefan; Steinmetz, Ralf; Baller, Gisa; Kalbe, Elke; El Moussaoui, Abdelkarim; Abels, Sven; Dienst, Susanne; Dornhoefer, Mareike; Fathi, Madjid	Adaptive procedure is not detailed
Lean Rehabilitative-Predictive Recommender Games for Seniors with Mild Cognitive Impairment: A Case Study	Lee, Chien-Sing; Yii, Wesly	Adaptive procedure is not detailed
