# Supplementary material for: Exploring the potential of artificial intelligence in individualized cognitive training: A systematic review
Source: PLoS One. 2025 Jun 17;20(6):e0316860. doi: 10.1371/journal.pone.0316860 (PMC12173387; doi:10.1371/journal.pone.0316860)
Supplement: Attachment — Submitted filename: response_to_reviewers.pdf [file pone.0316860.s004.pdf]

Response to Reviewers

Re: Manuscript ID PONE-D-23-33514

"Exploring the Potential of Artificial Intelligence in Individualized Cognitive Training: A Systematic Review"

PLOS ONE

Overview

We would like to begin by thanking the reviewers for their thoughtful feedback on our work. We have carefully addressed the majority of their comments to improve the clarity and quality of the paper. Regarding the general concern about the review being outdated, we would like to clarify that this was due to circumstances beyond our control. The paper was submitted in October 2023, and the review process experienced delays, including incorrect and AI-generated reviews. We have clearly stated in the manuscript that the literature search concluded in 2023 to ensure transparency for readers. Below, we address each comment individually.

Reviewer #1, comment #1

I think the use of AI should be mentioned much earlier in the Introduction. As it currently reads, it is not mentioned until page 6.

Our response #1.1

At the end of the first paragraph of the introduction, we have added sentences clearly stating that our focus is on existing AI strategies that enable individualized cognitive training (see lines [30-41]).

Reviewer #1, comment #2

Table 2 that reviews the papers should include age ranges. I think it may also be better to focus just on adult population studies rather than child, adult and older adults.

Our response #1.2

We have added the adult age ranges to Tables 2 and 3. While we understand the suggestion to focus on a specific population, the goal of this review is to examine all existing strategies for individualization. Due to the limited number of studies available, we included all population types to provide a comprehensive overview of the current state of the art.

Reviewer #1, comment #3

My other concern is that this review is now outdated (end date was June 2023) as the authors mention there is a huge increase in literature regarding this topic. If this is the case then the review may need updating to include at least another year.

Our response #1.3

We fully agree with the reviewer's concern. Unfortunately, this systematic review was submitted in October 2023, and the peer-review process was delayed due to initial incorrect reviews (unrelated to this paper) and a set of AI-generated reviews. Consequently, there was no additional time to search for, process, and incorporate more recent studies. We have clearly stated in the methods section when the literature search was conducted, ensuring that readers are informed about the search timeline.

Reviewer #2, comment #1

The major issue is that the period of review was from over 1.5 years ago, meaning by the time this is published, the most recent study referenced will be about two years old. In a rapidly growing field that is AI- and ML-based, this paper will be largely outdated at the time of publication.

Our response #2.1

Please refer to our response to Reviewer #1 for an explanation regarding the timeline and limitations in updating the review.

Reviewer #2, comment #2

It seems as though some more effort needs to be put into making sure the reader understands macro- and micro-adaptive strategies. The Figure (2) provided is not very intuitive for the macro-adaptive portion (a). Given that much of your argument and discussion revolves around a good understanding of these concepts, more care should be taken in driving home those points in the introduction.

Our response #2.2

We have revised the paragraph explaining micro- and macro-adaptive strategies to improve clarity (see specifically section [224-255]). Additionally, Figure 2 has been updated by removing unnecessary elements, such as the starting point square, to enhance its intuitiveness.

Reviewer #2, comment #3

This paper has just now come to review, and the screening for articles was stopped in February 2023 (over 1.5 years ago now). Given the fast-moving state of this field, especially pertaining to use of AI methods, this manuscript search will likely need to be updated, as it seems some delay has occurred between the time you wrote this paper and when it was submitted for review. The only issue here is that when someone cites your review, if published in 2024, it will be referring to literature reviewed from nearly two years prior to the publication of this article.

Our response #2.3

Please refer to our response to Reviewer #1 for an explanation regarding the timeline and limitations in updating the review.

Reviewer #2, comment #4

Reframe the question, "How effective are they in empirical CT studies?" in 3.4 Q4 to be a complete, standalone sentence (i.e. don't use the word "they", as the reader does not know what you're referring to).

Our response #2.4

We have reformulated the question in section 3.4 Q4 for clarity (see [363-365]).

Reviewer #2, comment #5

Grammar and punctuation issues with 3.5 Q5 (line 600; random capitalization, "have" instead of "has"; line 604 has a random use of the number "2" in a sentence instead of "two", as would be appropriate) – ensure a grammar check is done throughout the manuscript

Our response #2.5

We thank the reviewer for identifying these typos. A thorough grammar check has been completed throughout the manuscript.

Reviewer #2, comment #6

I feel like the results section could benefit from a figure that demonstrates what approaches were used by the most effective studies (e.g. those with positive outcomes likely due to their chosen CT approach and those which had ++ validation). If the reader comes to this paper wanting to know how best to design their ML-based, individualized, CT intervention they should easily be able to scroll to your results section and know the essentials of the procedures used most successfully to date.

Our response #2.6

We have created a new figure that integrates various results, including the impact of training and methodological quality, to highlight the approaches used by the most effective studies (see Figure 4: "Comparison of studies across single and multi-domain interventions in Cognitive Performance, Training Trajectories, and Subjective Experience.").

Reviewer #3, comment #1

The flowchart (fig.1) is unclear in some parts, e.g. in the sections 'studies excluded' and 'studies sought for retrieval' it would be helpful to specify the reasons. In the section 'studies included', 17 articles are reported, but it is not made clear that these are 19 studies. Please review and add these.

Our response #3.1

We have updated the flowchart to clarify the distinction between the number of articles included and the number of studies reviewed, as some articles contain multiple studies. Additionally, we specified the reasons for exclusions in the sections 'studies excluded' and 'studies sought for retrieval'.

Reviewer #3, comment #2

In the paragraph Sorting keys of AI techniques for content adapting to learner's capabilities the authors report that AI techniques can be classified into four main families, yet they only list three of them. Please make this information consistent.

Our response #3.2

We have revised this section to ensure consistency and clarity in the classification of AI techniques (see lines [224-255]).

Reviewer #3, comment #3

Please check that all acronyms in the paper are spelled out in full at least once, e.g. in the paragraph Evaluation of AI techniques the acronym NFT is not specified.

Our response #3.3

We conducted a thorough check to ensure all acronyms are defined at least once throughout the paper. The acronym NFT was spelled out on first use in the "Introduction" (line 96).

Reviewer #3, comment #4

The table 5 only shows 9 out of 19 studies, no reason is specified. Please clarify this point.

Our response #3.4

Table 5 excludes studies with non-comparative designs, as these automatically receive the lowest grade. We have added this clarification in the table description to ensure transparency.

Adolphe Maxime,
on behalf of all authors
