# Supplementary material for: Exploring the potential of artificial intelligence in individualized cognitive training: A systematic review
Source: PLoS One. 2025 Jun 17;20(6):e0316860. doi: 10.1371/journal.pone.0316860 (PMC12173387; doi:10.1371/journal.pone.0316860)
Supplement: Attachment — Submitted filename: PLOSone_Review_9.5.24.docx [file pone.0316860.s003.docx]

**PLOS-One Review Sept 2024**

**Introduction**

- It seems as though some more effort needs to be put into making sure the reader understands macro- and micro-adaptive strategies. The Figure (2) provided is not very intuitive for the macro-adaptive portion (a). Given that much of your argument and discussion revolves around a good understanding of these concepts, more care should be taken in driving home those points in the introduction.

**Materials**

- This paper has just now come to review, and the screening for articles was stopped in February 2023 (over 1.5 years ago now). Given the fast-moving state of this field, especially pertaining to use of AI methods, this manuscript search will likely need to be updated, as it seems some delay has occurred between the time you wrote this paper and when it was submitted for review. The only issue here is that when someone cites your review, if published in 2024, it will be referring to literature reviewed from nearly two years prior to the publication of this article.

**Results**

- Reframe the question, “How effective are they in empirical CT studies?” in 3.4 Q4 to be a complete, standalone sentence (i.e. don’t use the word “they”, as the reader does not know what you’re referring to).
- Grammar and punctuation issues with 3.5 Q5 (line 600; random capitalization, “have” instead of “has”; line 604 has a random use of the number “2” in a sentence instead of “two”, as would be appropriate) – ensure a grammar check is done throughout the manuscript
- I feel like the results section could benefit from a figure that demonstrates what approaches were used by the most effective studies (e.g. those with positive outcomes likely due to their chosen CT approach and those which had ++ validation). If the reader comes to this paper wanting to know how best to design their ML-based, individualized, CT intervention they should easily be able to scroll to your results section and know the essentials of the procedures used most successfully to date.
